# An Event Related Potentials Study of Semantic Coherence Effect during Episodic Encoding in Schizophrenia Patients

**DOI:** 10.1155/2018/8501973

**Published:** 2018-01-01

**Authors:** Lâle Battal Merlet, Alain Blanchet, Hazlin Lockman, Milena Kostova

**Affiliations:** ^1^Université Paris Lumières, Université Paris 8, Laboratoire de Psychopathologie et Neuropsychologie, EA 2027, Saint-Denis, France; ^2^Psychological Medicine Department, University Malaya, Kuala Lumpur, Malaysia

## Abstract

The objective of this electrophysiological study was to investigate the processing of semantic coherence during encoding in relation to episodic memory processes promoted at test, in schizophrenia patients, by using the N400 paradigm. Eighteen schizophrenia patients and 15 healthy participants undertook a recognition memory task. The stimuli consisted of pairs of words either semantically related or unrelated to a given category name (context). During encoding, both groups exhibited an N400 external semantic coherence effect. Healthy controls also showed an N400 internal semantic coherence effect, but this effect was not present in patients. At test, related stimuli were accompanied by an FN400 old/new effect in both groups and by a parietal old/new effect in the control group alone. In the patient group, external semantic coherence effect was associated with FN400, while, in the control group, it was correlated to the parietal old/new effect. Our results indicate that schizophrenia patients can process the contextual information at encoding to enhance familiarity process for related stimuli at test. Therefore, cognitive rehabilitation therapies targeting the implementation of semantic encoding strategies can mobilize familiarity which in turn can overcome the recollection deficit, promoting successful episodic memory performance in schizophrenia patients.

## 1. Introduction

Cognitive dysfunctioning is a core feature in schizophrenia [1], with episodic memory being one of the most impaired cognitive areas in this pathology [2, 3]. In the context of dual process models, familiarity and recollection are two distinct processes within episodic memory [4–6]. Familiarity refers to the judgment of prior occurrence, while recollection is accompanied by contextual or associative information related to the stimulus. Both processes are altered in schizophrenia [7–9]. In spite of this alteration, schizophrenia patients benefit from explicit semantic strategies provided at encoding which promote successful memory performance during test [10, 11]. However, studies show that while, in healthy controls, episodic memory enhancement can be related to an increase in both familiarity and recollection estimates [5, 12–14], in schizophrenia patients, this enhancement is linked to an increase in familiarity, alone [15–17].

For example, in a recent behavioral study [17], we designed an associative recognition memory task to test the effect of semantic coherence between a category name and a word pair on familiarity and recollection processes in schizophrenia. We used the process dissociation procedure (PDP, [4]) to calculate the respective parts of these processes contributing to the overall recognition performance. During the study phase, pairs of words either semantically related or unrelated to a given category name were deeply encoded [18]. During the test, the results indicated an enhancement of episodic memory performance for semantically related word pairs compared to semantically unrelated ones in both healthy controls and schizophrenia patients. In healthy controls, this enhancement was linked to an increase in recollection estimates, whereas, in schizophrenia patients, semantic coherence led to increased familiarity. Thereby, this pattern of the results obtained during the test must be in relation to different strategies employed during encoding by the two groups of participants. In other words, even under the same explicit encoding instructions and having similar categorization performances during the study, schizophrenia patients should have used different encoding strategies compared to the ones employed by healthy individuals, which in turn should have led to an increase in familiarity estimates alone in this group. Literature indicates that, in associative recognition memory tasks, interitem binding mechanisms can lead to successful recollection process [19]. Accordingly, the semantic relationship between the two items within a pair, when processed at encoding, should create a tie which facilitates successful recollection at test. In schizophrenia patients, binding different components of the information to form a relational memory representation seems to be affected [20, 21]. Consequently, schizophrenia patients may not process the interitem semantic relationship that we will call “internal semantic coherence” (within the word pair), while they may be receptive to the semantic coherence between the context (category name) and the stimulus that we will call here “external semantic coherence.” The objective of the present study was precisely to test if schizophrenia patients process both internal and external semantic coherence during encoding.

For the investigation of the strategies adopted during encoding phase, event related potentials (ERPs) could provide invaluable information because of their high temporal resolution allowing the real-time tracking of underlying neurocognitive processes in use. The N400 paradigm is indeed the best candidate to pinpoint the detection of internal and external semantic coherence during encoding. The N400 corresponds to a negative-going component which peaks around 300–500 ms after the stimulus onset. Its amplitude is modulated by factors such as semantic or associative relationship between the target stimulus and the prime [22]. The N400 amplitude decreases when a prime activates semantic properties of the target, indicating the overlap between the activated semantic features by the prime and the characteristics of the target item. Thus, the N400 effect corresponds to the difference between N400 amplitudes evoked in two different experimental conditions (e.g., related versus unrelated targets) (for a review, see [23]). Thereby, we hypothesized that, during encoding, schizophrenia patients would not process the internal but only the external semantic coherence. By contrasting the N400 component amplitudes elicited by semantically related words versus semantically unrelated ones, for the second item of the word pairs presented sequentially during encoding, we should find an N400 internal coherence effect (i.e., reduced N400 amplitudes when the second word of the pair is related to the first one compared to unrelated words) in healthy controls, but not in schizophrenia patients. However, we should observe in both groups an N400 external coherence effect (i.e., reduced N400 amplitudes when the first word of the pair is a category exemplar compared to nonexemplars).

Studies investigating episodic memory processes by the means of ERPs compared the ERP patterns generated by the presentation of old stimuli with the ones induced by the presentation of new items during the test to individualize the ERP correlates of familiarity and recollection. Two ERP old/new effects (difference wave between hits and correct rejections) have been individualized as indices of familiarity and recollection processes ([24–26]). The mid-frontal ERP old/new effect is a negative-going pattern (FN400) elicited around 300–500 ms after the presentation of the stimulus with maximum amplitude over frontal sites. It corresponds to an increased negativity for new items compared to the old ones. The parietal old/new effect is a left parietal positivity appearing around 500–800 ms after the stimulus onset with maximum amplitude over left temporoparietal sites. It refers to the positivity for the old items when the amplitudes of the P600 (or LPC for late positive component) generated for correctly recognized old and new items are contrasted. The FN400 effect is not sensitive to the retrieval of the specific associative or contextual information related to the study, while parietal old/new effect is modulated by the recollection of such information, in addition to being able to differentiate old from new [27, 28].

The effect of what we call here external semantic coherence on episodic memory processes was investigated in healthy controls by the means of ERPs [12]. The results indicated a familiarity based retrieval advantage. This effect has not yet been investigated in schizophrenia patients by using electrophysiological data. Consequently, in the present study, we aimed to investigate both encoding and retrieval stages in an associative recognition memory task in schizophrenia patients, using deep encoding and manipulating the semantic coherence of to be learned stimuli. In addition to our hypotheses for internal and external N400 effects during the study phase, we also believed that, during the test phase, the increase in correct response rates for semantically related word pairs would be accompanied by an FN400 effect in schizophrenia patients, while in healthy controls both early mid-frontal and late parietal old/new effects would be observed for the related stimuli.

Finally, to the best of our knowledge, no electrophysiological study has established an explicit link between the ERP correlates of encoding strategies (e.g., processing of external and internal semantic coherence) used by schizophrenia patients at study and the ERP indices of episodic memory processes promoted at test. Consequently, by putting together our hypotheses concerning the study phase with those related to the test, we thought that in schizophrenia patients, the N400 external coherence effect expected during encoding would be followed by an FN400 old/new effect for the related stimuli during test, whereas, in healthy controls, the N400 external and internal coherence effects predicted at encoding would be followed by both early mid-frontal and late parietal old/new effects for the related stimuli during the test phase.

## 2. Methodology

### 2.1. Participants

This study was carried out in accordance with the latest declaration of Helsinki. All the participants gave informed written consent. Twenty-four schizophrenia patients between the ages of 22 and 59 years were recruited in the day care unit of University Malaya Psychological Medicine Department in Kuala Lumpur, Malaysia. The diagnosis of schizophrenia was established according to DSM IV [29] criteria by the psychiatrists of the hospital who were blinded to the aims and objectives of the study. All the patients were stabilized. They were under atypical neuroleptic treatment before and during the study. The chlorpromazine equivalents of the medication are presented in the Table 1.

The patients had a psychiatric interview (the structured interview for PANSS; SCI-PANSS [30]) with the psychiatrist of the day care. They were assessed by the means of the positive and the negative syndrome scale for schizophrenia (PANSS; [31]). Sixteen healthy volunteers between the ages of 22 and 59 years were also recruited from the community as the control group. They had a clinical interview with the psychiatrist of the daycare. They were free of any detectable psychiatric or neurological pathology during the time of their inclusion. All the participants were assessed by using the verbal subscale of Kaufman Brief Intelligence Test (KBIT-2) [32].

Among participants, two schizophrenia patients and one healthy individual did not complete the protocol and consequently were excluded from statistical analyses. Two more patients were excluded because of technical issues, while two other schizophrenia patients had too poor performances in the semantically unrelated condition of the test that they could not produce any valid electrophysiological segments for the ERPs and were consequently excluded. As a result, all statistical analyses were conducted with eighteen schizophrenia patients and fifteen healthy volunteers. The participants were paid the equivalent of 30 US§ each for their contribution to the study.

The demographic and clinical data in the two groups are presented in the Table 1.

### 2.2. Material

**Figure 1:**
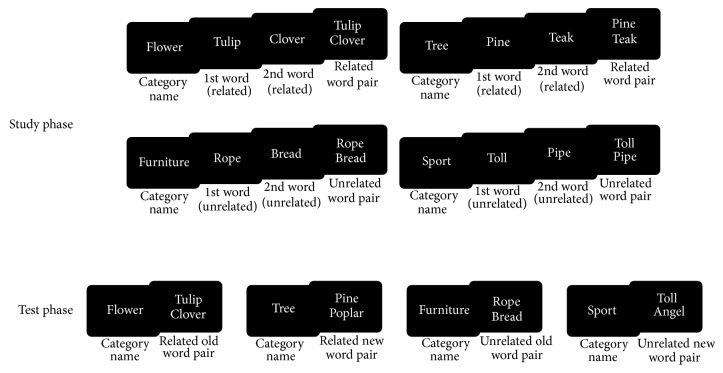
Examples of stimuli during the study and test phases.

The experimental material consisted of a list of 512 words containing nouns of four to six letters with a frequency of 1 to 100 occurrences per million [33]. For the study phase, we created a list of 224 word pairs. From these pairs, 96 pairs contained two words related to a given semantic category (also related to each other, being exemplars of this category), 32 pairs contained only one related word and used as fillers, and 96 other pairs consisted of two unrelated words (unrelated to the given category and unrelated to each other). Related words were chosen within 24 different categories such as mammal or beverage. Unrelated words did not belong to any of these categories. These word pairs were then distributed into four blocks containing each a study list of 56 pairs (24 pairs with two related words, 8 pairs with one related word, and 24 pairs with two unrelated words) and a test list of 48 pairs (20 pairs with two related words, 8 pairs with one related word, and 20 pairs with two unrelated words). Twenty-four word pairs of the study phase became intact word pairs during the test (8 pairs with two related words, 8 pairs with one related word, and 8 pairs with two unrelated words). Sixteen words pairs of the study phase were rearranged to obtain 8 recombined pairs during test (4 pairs with two related words and 4 pairs with two unrelated words). Hence, in a recombined word pair, there are two old items that were presented in two previously encountered pairs. These old items have now been put together to create a different pair. Consequently, the items are old but the pair itself is new. Finally, sixteen pairs of the study were used to create new word pairs during test by combining the first word of the each pair with a nonstudied word (8 pairs with two related words and 8 pairs with two unrelated words). Word pairs with one related word (first word or second word related to the given category name) were used to encourage the treatment of both words in a pair during the study to decide if one word, two words, or none of the words belong to the given category name, without jumping to the conclusion as soon as the participants see the first word. Thereby, one related word pairs and recombined word pairs were used as fillers and discarded from statistical analyses. The rearrangement of word pairs was realized within each category. Consequently, the category name associated with a given word was the same during the study and the test. Examples of stimuli during the study and test phases are presented in the Figure 1.

We controlled the word pairs' mean frequencies in different conditions at study and test phases. During the study phase, the mean frequencies for related (two words related) and unrelated (two words unrelated) word pairs did not show any significant differences (mean frequency for unrelated word pairs = 21.03 ± 0.10, mean frequency for related word pairs = 18.75 ± 0.13; *F*(1,190) = 1.78; *P* = 0.184;* Partial*  **η** ^2^ = 0.00).

During the test, repeated measures ANOVA with coherence (related, unrelated) by Pair (intact, new) did not show any main effects neither interactions between these factors (*F*s < 3.2, *P* > 0.08; related old = 19.5 ± 0.11; related new = 16.5 ± 0.14; unrelated old = 22.1 ± 0.10; unrelated new = 18.5 ± 0.10).

### 2.3. Procedure

The experiment was implemented using E-Prime 1.2 (Psychology Software Tools, https://www.pstnet.com). The stimuli were presented in white on a black screen using lowercase Courier New 18-point font. The participants were seated comfortably at a viewing distance of approximately 75 cm from the screen. The study and the test phases started with a fixation cross of 750 ms in the middle of the screen followed by a blank of 250 ms. The category name was then presented for 1500 ms before being replaced with a blank of 450 ms. After this, during the study phase, the first word of the pair was displayed for 450 ms followed by a blank of 450 ms and by the second word of the pair for 450 ms. The two words of the pair were then presented as one word above the other in the middle of the screen and the participants were invited to answer if one word, two words, or none of the words belonged to the given category name by pressing on the keyboard (0, 1, or 2). Following the answer, they were given a true/false feedback and the word pair was displayed again for 450 ms.

**Figure 2:**
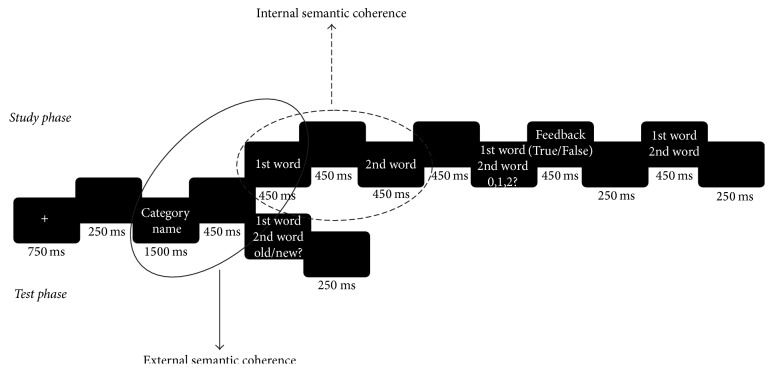
Procedure at study and test phases.

During the test, the word pair was displayed 450 ms after the presentation of the category name and the participants were invited to make old/new judgments by using the mouse. The intact word pairs were to be considered as old while recombined and new word pairs were expected to be judged as new, since it was to decide if the two words within a pair were presented together during the study. When an answer was made the screen became blank for 250 ms before the next trial. The participants were instructed to answer as fast and accurate as they could. The presentation of the new word within the word pair (first word or second word is new) in related and unrelated conditions were counterbalanced within participants. The presentation of the stimuli within each block was randomized. A study/test trial was conducted at the beginning of the protocol to familiarize the participants with the procedure. The procedure is presented in Figure 2.

### 2.4. Behavioral Measures

The correct response rates for the study (0, 1, or 2 words related to a given category) and test phases (old/new) were calculated for each participant and each condition. The discrimination accuracy between old and new items (*d*′) and decision criterion (*C*) indices were also calculated from hits (“old” responses for intact pairs) and false alarms (“old” responses for new word pairs) for related and unrelated conditions. *d*′ = *z* (Hits)  −  *z* (False alarms). When hits equal false alarms, *d*′ becomes zero. The larger *d*′, the better indeed the discrimination accuracy. *C* = −(*z* (Hits) + *z* (False alarms))/2. For *C* indices, negative values indicate a liberal decision criterion (more willing to respond “old”), while values above zero correspond to a conservative bias (less willing to respond “old”) [34].

### 2.5. Electrophysiological Recording and Analyses

The electroencephalographic activity (EEG) was recorded from the scalp through 27 electrodes implemented on an electrode cap (EasyCap©; Herrsching-Breitbrunn, Germany) following the extended international 10/20 system. AFz served as the ground electrode. A common reference was used during the acquisition, but two linked earlobe electrodes were included in the recording montage for offline recalculation of the reference. Eye movements were controlled by the two electrodes placed one at the outer canthus and the other above the right eye. For all electrodes the impedance was maintained below 10 kohm. The signal was amplified using a QuickAmp amplifier (http://www.brainproducts.com/index.php). The EEG was recorded with a bandpass filter of 0.01–100 Hz and digitized at 500 Hz. The data were digitally filtered at a bandpass of 0.80–12 Hz. The duration of each epoch was 1100 ms, including a prestimulus baseline of 100 ms. Artifacts were reduced by correcting for eye movements using the regression-based approach (Gratton, Coles, and Donchin algorithm, 1983) and by rejecting epochs with voltages exceeding ±100 *μ*V in any EEG channel. ERPs were extracted from the first and the second words of each pair at encoding and from old and new word pairs at test. The ERPs evoked by correct answers were averaged for each subject and each condition. Only 0.05% of epochs were lost due to artifacts. Based on the visual inspection of the plots, for FN400 and N400 components, the mean amplitudes (*μ*V) were measured for 300–600 ms after stimulus time window, while for the LPC component 500–800 ms after stimulus time window was used.

### 2.6. Statistical Analyses

Statistical analyses were carried out using PASW Statistics 19 (SPSS Inc., Chicago, IL, USA). The mean rates presented in the text, tables, and figures are provided with standard deviations. The sociodemographic and clinical characteristics of the two groups of participants were analyzed by using *t*-tests and chi2 tests. For the correct response rates during the study phase, a one-way ANOVA was performed with group (control, schizophrenia) set as the between-subjects factor. The discrimination accuracy and the decision criterion rates were analyzed by the means of repeated measures ANOVAs with group set as the between-subjects factor and the coherence (related, unrelated) set as the within-subjects factor. For the analyses of the correct response rates during the test, repeated measures ANOVAs were performed with group set as the between-subjects factor, and old/new (old, new) and coherence (related, unrelated) set as the within-subjects factors.

Repeated measures ANOVA with group set as the between-subjects factor, coherence (related, unrelated), and electrodes (C3, CZ, C4, CP1, CP2, P3, PZ, and P4) set as the within-subjects factor, and the mean amplitudes set as the dependent variable for the 300–600 ms time window after the stimulus onset was run to analyze the processing of internal and external semantic coherence, respectively, during the study phase. For the test phase, the 300–600 ms time window after the stimulus onset was analyzed by the means of repeated measures ANOVAs, with group as between-subjects factor, coherence (related, unrelated), old/new (old, new), hemisphere (left, right), and electrodes (FP1, F3, and FC1 for the left; FP2, F4, and FC2 for the right hemisphere) set as the within-subjects factors on the mean amplitudes set as the dependent variable. Finally, the 500–800 ms time window after the stimulus onset was analyzed by a repeated measures ANOVA with group set as the between-subjects factor, coherence (related, unrelated), old/new (old, new), hemisphere (left, right), and electrodes (C3, CP1, and P3 for the left; C4, CP2, and P4 for the right hemisphere) set as the within-subjects factors on the mean amplitudes set as the dependent variable. The probability levels for *F*-tests were calculated. When appropriate, the Greenhouse-Geisser correction of degrees of freedom for repeated measures was applied. Planned comparisons were done for the analyses of the simple effects. The alpha level was set at 0.05.

Finally, Pearson correlation coefficients were calculated between demographic (years of education) and clinical variables (PANSS negative, positive, and general psychopathology scores, duration of illness, and chlorpromazine equivalents of medication) and behavioral (categorization performance at study, correct response rates for old related, old unrelated, new related, and new unrelated word pairs at test) and electrophysiological measurements (N400 external and internal coherence effects (average amplitudes at the electrodes C3, CZ, C4, CP1, CP2, P3, PZ, and P4), FN400 old/new effect (average amplitudes at the electrodes FP1, FP2, F3, F4, FC1, and FC2), and LPC old/new effect (average amplitudes at the electrodes C3, C4, CP1, CP2, P3, and P4)) in order to control the effect of demographic and clinical variables on experimental measurements. Also, to bring further understanding to the relationship between encoding strategies at study and memory processes at test, correlations between N400 external and internal coherence effects and FN400 and LPC old/new effects were calculated.

## 3. Results

### 3.1. Sociodemographic and Clinical Data

The control group's education level was higher than that of the patient group (*t*(1,31) = 2.4; *P* = 0.022). There was however no significant difference between the verbal IQ levels in the two groups (*t*(1,31) = −0.20; *P* = 0.984). There was no difference between the groups concerning the age (*t*(1,31) = 1.45; *P* = 0.156), and the gender compositions of the groups were similar (*χ* ^2^ = 0.423, d.f. = 1, *P* = 0.515).

### 3.2. Behavioral Results

#### 3.2.1. Study Phase

During the study phase, there was no significant difference in the correct response rates between the two groups (healthy controls = 0.77 ± 0.07; schizophrenia = 0.78 ± 0.11; *F*(1,31) = 0.50; *P* = 0.825;* Partial*  **η** ^2^ = 0.00).

#### 3.2.2. Test Phase

*(1) Old/New Discrimination Accuracy and Decision Criterion*. The ANOVA run on group by coherence (related, unrelated) with the old/new discrimination accuracy (*d*′) rates set as the dependent variable showed a main effect of group (*F*(1,31) = 8.17; *P* = 0.008;* Partial*  **η** ^2^ = 0.20). Healthy controls had higher discrimination accuracy rates (*d*′ = 2.04 ± 0.71) than schizophrenia patients (*d*′ = 1.36 ± 0.66). There was also a main effect of coherence (*F*(1,31) = 24.68; *P* < 0.001;* Partial*  **η** ^2^ = 0.44) with higher discrimination accuracy rates for related (*d*′ = 2.05 ± 0.92) than unrelated word pairs (*d*′ = 1.34 ± 0.79). No interaction was found between group and coherence (*F*(1,31) = 0.37; *P* = 0.548;* Partial*  **η** ^2^ = 0.01).

The ANOVA performed on group by coherence with decision criterion (*C*) rates set as the dependent variable revealed that there was no effect of group (*F*(1,31) = 0.73; *P* = 0.396;* Partial*  **η** ^2^ = 0.02; controls: *C* = −0.04 ± 0.29; schizophrenia = 0.06 ± 0.42), but a main effect of coherence (*F*(1,31) = 11.52; *P* = 0.002;* Partial*  **η** ^2^ = 0.27; related word pairs: *C* = −0.24 ± 0.56; unrelated word pairs: *C* = 0.25 ± 0.55), and a coherence by group interaction (*F*(1,31) = 4.23; *P* = 0.048;* Partial*  **η** ^2^ = 0.12). In the control group, the difference between *C* indices for related word pairs and those for unrelated word pairs did not reach the significance level (related word pairs: *C* = −0.14 ± 0.29, unrelated word pairs: *C* = 0.04 ± 0.40; *P* = 0.081), while in the schizophrenia group the difference was significant (related word pairs: *C* = −0.31 ± 0.71, unrelated word pairs: *C* = 0.44 ± 0.60; *P* = 0.006), indicating a more conservative decision criterion for unrelated word pairs than related ones in schizophrenia patients. There was no group difference for related word pairs (*P* = 0.347), while, for unrelated word pairs, schizophrenia patients had higher rates than healthy controls (*P* = 0.032), suggesting a more conservative decision criterion in this condition.

*(2) Correct Response Rates*. Mean correct response rates and standard deviations during the test phase for each word pair and coherence condition in the two groups are presented in the Table 2.

A 2X2X2 repeated measures ANOVA performed on the correct response rates revealed a main effect of group (*F*(1,31) = 9.61; *P* = 0.004;* Partial*  **η** ^2^ = 0.23; healthy controls = 0.81 ± 0.11; schizophrenia patients = 0.69 ± 0.14). There was a main effect of coherence (*F*(1,31) = 23.66; *P* < 0.001;* Partial*  **η** ^2^ = 0.43) revealing that related word pairs induced higher scores compared to unrelated ones. There was also a significant old/new by coherence interaction (*F*(1,31) = 12.38; *P* = 0.001;* Partial*  **η** ^2^ = 0.28) and a group by old/new by coherence interaction (*F*(1,31) = 4.13; *P* = 0.051;* Partial*  **η** ^2^ = 0.11) at strong tendency level.

*The Control Group*. The analyses revealed a main effect of coherence (*F*(1,14) = 15.38; *P* = 0.002;* Partial*  **η** ^2^ = 0.52), and an old/new by coherence interaction (*F*(1,14) = 5.48; *P* = 0.034;* Partial*  **η** ^2^ = 0.28). Breaking down this interaction showed that semantic coherence induced higher scores for old word pairs (*P* < 0.001), but for new word pairs semantic coherence did not affect the scores (*P* = 0.447).

*The Schizophrenia Group*. The analyses showed a main effect of coherence (*F*(1,17) = 10.42; *P* = 0.005;* Partial*  **η** ^2^ = 0.38) and an old/new by coherence interaction (*F*(1,17) = 10.14; *P* = 0.005;* Partial*  **η** ^2^ = 0.37), revealing that schizophrenia patients had the same pattern as the controls with higher scores for related than unrelated old word pairs (*P* < 0.001), while, for new word pairs, the effect of semantic coherence was not significant (*P* = 0.111).

*Old Pairs*. There was a main effect of group (*F*(1,31) = 7.55; *P* = 0.010;* Partial*  **η** ^2^ = 0.19), an effect of coherence (*F*(1,31) = 31.84; *P* < 0.001;* Partial*  **η** ^2^ = 0.50), and a group by coherence interaction at strong tendency level (*F*(1,31) = 3.95; *P* = 0.055;* Partial*  **η** ^2^ = 0.11), indicating that schizophrenia patients had lower scores than healthy controls for unrelated word pairs (*P* = 0.011), while for related ones the scores were similar in the two groups (*P* = 0.189).

*New Pairs*. Schizophrenia patients had similar scores to those obtained by healthy controls (*F*(1,31) = 2.70; *P* = 0.110;* Partial*  **η** ^2^ = 0.08). There was no effect of coherence (*F*(1,31) = 1.21; *P* = 0.278;* Partial*  **η** ^2^ = 0.03). The group by coherence interaction was not significant (*F*(1,31) = 3.02; *P* = 0.092;* Partial*  **η** ^2^ = 0.08).

### 3.3. Electrophysiological Results

#### 3.3.1. Study Phase

*(1) N400 External Coherence Effect*. A 2X2X8 repeated measures ANOVA on the amplitudes of the ERPs evoked by the first item of the word pairs showed no significant effect of group (*F*(1,31) = 3.28; *P* = 0.080;* Partial*  **η** ^2^ = 0.09). There was a main effect of coherence (*F*(1,31) = 7.83; *P* = 0.009;* Partial*  **η** ^2^ = 0.20) with unrelated word pairs inducing more negative amplitudes compared to related word pairs (related = 0.60 ± 0.75; unrelated = 0.39 ± 0.60). There was also a main effect of electrode (*F*(7,217) = 10.42; *P* < 0.001;* Partial*  **η** ^2^ = 0.25) and a Group by Electrode interaction (*F*(7,217) = 2.96; *P* = 0.042;* Partial*  **η** ^2^ = 0.08). Other interactions did not reach the significance level (All *F*s < 2.26, *P*s > 0.140).

*(2) N400 Internal Coherence Effect*. A 2X2X8 repeated measures ANOVA on the amplitudes of the ERPs evoked by the second item of the word pairs indicated that there was no effect of group (*F*(1,31) = 0.00; *P* = 0.999;* Partial*  **η** ^2^ = 0.00), but a main effect of coherence (*F*(1,31) = 11.12; *P* = 0.002;* Partial*  **η** ^2^ = 0.26), a main effect of electrode (*F*(7,217) = 22.20; *P* < 0.001;* Partial*  **η** ^2^ = 0.41), and a group by coherence by electrode interaction (*F*(7,217) = 2.69; *P* = 0.041;* Partial*  **η** ^2^ = 0.08).

In the control group there was a main effect of coherence (*F*(1,14) = 11.13; *P* = 0.005;* Partial*  **η** ^2^ = 0.44). Unrelated word pairs induced more negative amplitudes compared to related ones (related = 0.23 ± 0.78; unrelated = −0.20 ± 0.60). There was also a coherence by electrode interaction (*F*(7,98) = 4.13; *P* = 0.013;* Partial*  **η** ^2^ = 0.22). The analysis of this interaction showed that the effect of coherence was significant for the electrodes CZ (*P* = 0.002), C4 (*P* = 0.008), CP1 (*P* = 0.015), CP2 (*P* = 0.002), PZ (*P* = 0.007), and P4 (*P* = 0.002).

In the patient group, the effect of coherence was not significant (*F*(1,17) = 1.87; *P* = 0.189;* Partial*  **η** ^2^ = 0.09; related = 0.10 ± 1.05; unrelated = −0.07 ± 0.87). There was no coherence by electrode interaction either (*F*(7,119) = 1.73; *P* = 0.183;* Partial*  **η** ^2^ = 0.09).

**Figure 3:**
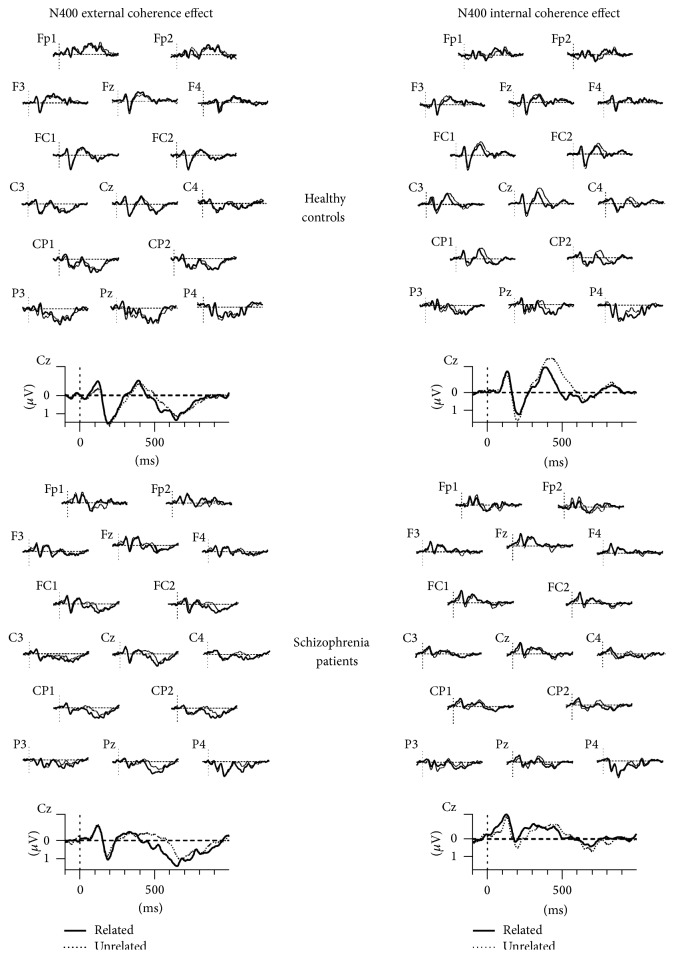
Grand average ERPs at fifteen representative electrode sites for the first (external semantic coherence) and the second words (internal semantic coherence) of word pairs in related versus unrelated conditions during the study phase in healthy controls and schizophrenia patients.

Grand average ERPs for the first (external semantic coherence) and the second word (internal semantic coherence) of word pairs in related versus unrelated conditions during the study phase in healthy controls and schizophrenia patients are represented in Figure 3.

#### 3.3.2. Test Phase

*(1) Mid-Frontal Old/New Effect (FN400)*. A 2X2X2X2X3 repeated measures ANOVA performed on the amplitudes of the ERPs evoked by correctly recognized items revealed no main effect of group (*F*(1,31) = 0.64; *P* = 0.428;* Partial*  **η** ^2^ = 0.02). There was a main effect of hemisphere (*F*(1,31) = 4.35; *P* = 0.045;* Partial*  **η** ^2^ = 0.12) and a coherence by old/new interaction (*F*(1,31) = 4.32; *P* = 0.046;* Partial*  **η** ^2^ = 0.12), showing that for related word pairs there was an old/new effect (*F*(1,32) = 4.86; *P* = 0.035;* Partial*  **η** ^2^ = 0.13) with new pairs inducing more negative amplitudes in comparison to old ones (old = −0.15 ± 1.32; new = −0.51 ± 1.01). For unrelated word pairs, the old/new effect was not significant (*F*(1,32) = 1.39; *P* = 0.246;* Partial*  **η** ^2^ = 0.04; old = −0.42 ± 1.20; new = −0.16 ± 1.13).

**Figure 4:**
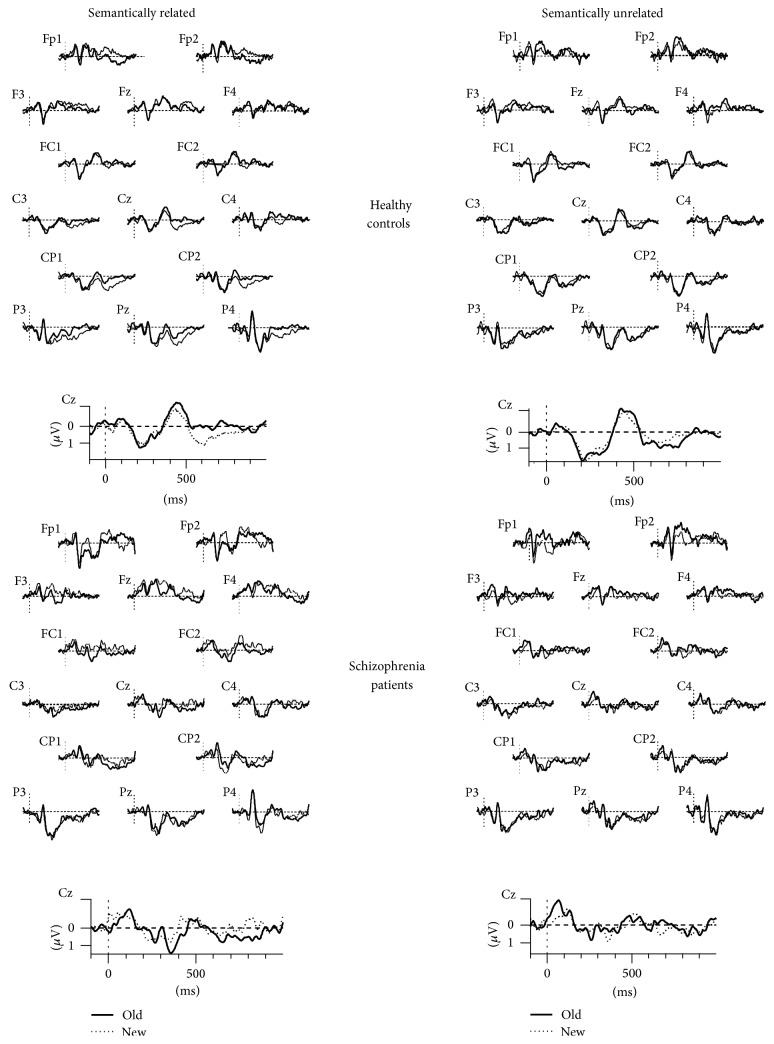
Grand average ERPS at fifteen representative electrodes sites for correct recognition of old versus new word pairs in related and unrelated conditions during the test phase, in healthy controls and schizophrenia patients.

*(2) Parietal Old/New Effect (P600 or LPC)*. A2X2X2X2X3 repeated measures ANOVA performed on the amplitudes of the ERPs evoked by correctly recognized items showed no main effect of group (*F*(1,31) = 0.37; *P* = 0.545;* Partial*  **η** ^2^ = 0.01), but a main effect of hemisphere (*F*(1,31) = 5.23; *P* = 0.029;* Partial*  **η** ^2^ = 0.14), a main effect of electrode (*F*(2,62) = 6.34; *P* = 0.011;* Partial*  **η** ^2^ = 0.17), and a coherence by old/new by group interaction (*F*(1,31) = 4.44; *P* = 0.043;* Partial*  **η** ^2^ = 0.12). Further analyses of this triple interaction indicated that, in healthy controls, there was a main old/new effect (*F*(1,14) = 7.08; *P* = 0.019;* Partial*  **η** ^2^ = 0.33) and a significant coherence by old/new interaction (*F*(1,14) = 7.44; *P* = 0.016;* Partial*  **η** ^2^ = 0.34) indicating that for related word pairs the old/new effect was significant (*F*(1,14) = 19.70; *P* = 0.001;* Partial*  **η** ^2^ = 0.58; old = 0.08 ± 0.69; new = 0.73 ± 0.73), while for unrelated word pairs it was not significant (*F*(1,14) = 0.01; *P* = 0.910;* Partial*  **η** ^2^ = 0.00; old = 0.51 ± 0.71; new = 0.49 ± 0.83). In the schizophrenia group, there were no main effects, neither any interactions (All *F*s < 0.50). Grand average ERPs for correct recognition of old versus new word pairs in related and unrelated conditions during the test phase in healthy controls and schizophrenia patients are shown in Figure 4.

#### 3.3.3. Correlations

There was a negative correlation between PANSS Negatives scores and behavioral results for old unrelated items (*r* = −0.48, *P* = 0.043), while years of education were positively correlated with correct response rates for new related word pairs (*r* = 0.44; *P* = 0.010). In healthy controls, the N400 external coherence effect amplitudes were correlated with the parietal old/new effect amplitudes (*r* = 0.53, *P* = 0.043), whereas in schizophrenia patients the N400 external coherence effect amplitudes had correlations with the FN400 old/new effect amplitudes (*r* = 0.48, *P* = 0.042).

## 4. Discussion

The aim of this study was to investigate the encoding and retrieval phases in an associative recognition memory task in schizophrenia patients by the means of ERPs. We manipulated the semantic coherence of to be learned stimuli and expected semantically related word pairs to induce higher recognition rates compared to unrelated word pairs in both healthy controls and schizophrenia patients. We hypothesized that, during encoding, the electrophysiological data obtained in schizophrenia patients would indicate an N400 external coherence but a lack of an N400 internal coherence effect between the related and unrelated conditions. We also believed that this lack of N400 internal coherence effect in schizophrenia patients would be accompanied by a lack of parietal old/new effect during retrieval. However, we thought there would be an early mid-frontal old/new effect for semantically related stimuli in the patient group. As expected, the behavioral results showed an increase of correct response rates for semantically related stimuli in comparison to semantically unrelated stimuli in both groups. The electrophysiological data indicated that, as hypothesized, healthy controls, but not schizophrenia patients, presented the N400 internal coherence effect between the related and unrelated conditions during encoding, whereas the N400 external coherence effect was present in both groups. Test phase data showed an early mid-frontal negativity for new word pairs compared to old word pairs, accompanied by a late positivity for new word pairs compared to old ones, for the related condition in healthy controls alone, while, in schizophrenia patients, only early mid-frontal old/new effect was observed for related stimuli.

### 4.1. Behavioral Data

The behavioral results obtained during the study phase indicated that schizophrenia patients encoded the stimuli as successfully as healthy controls. During the test, old/new discrimination accuracy indices showed that semantic coherence helped both groups to increase their performances. Decision criterion indices showed that schizophrenia patients had more conservative rates for the unrelated condition compared to the related one. In the unrelated condition, their decision criterion rates were more conservative compared to those obtained by healthy controls, too. In the related condition, there was, however, no difference between the criterion rates obtained by the two groups. Semantic coherence helped schizophrenia patients to normalize their decision criterion rates that were otherwise too conservative. Concerning the false alarm rates, the results indicated that semantic coherence did not affect the performance in a significant way, which is in accordance with studies showing that schizophrenia patients do not make more false alarms than healthy controls in general [35]. In this study carried out by Elvevag and collaborators, patients made even fewer false alarms than controls in the condition of semantically related lures. In our study, the difference between the performances of the two groups did not reach the significance level. This is also in accordance with the results obtained by Moritz and collaborators [36], showing no difference between the patients and the controls on false recognition rates of related and unrelated lures. In addition, in our study, even though new word pairs, when semantically related, induced lower recognition rates than semantically unrelated ones in the patients group, the difference was not significant. Furthermore, in our previous behavioral study [17], false alarm rates obtained by schizophrenia patients were globally similar to those found in healthy controls, too. As a consequence, in the present study, semantic coherence induced a real enhancement in recognition performances in both healthy controls and schizophrenia patients, without affecting the false alarm rates.

There was a negative correlation between the PANSS negative scale scores and the correct response rates for old unrelated items. The association between negative symptoms and the results of cognitive measurements suggested by this negative correlation corroborates the findings of literature showing the relationship between negative symptoms and cognitive deficit in general [37] and the link between negative symptoms and verbal episodic memory dysfunctioning in particular [16, 38]. Correlation analyses' results also suggest that even though schizophrenia patients having more negative symptoms exhibit lower recognition scores for old items, they seem to benefit from the positive effect of semantic coherence on episodic memory performance, since no correlations exist between the PANSS negative scores and the performances for old items when related.

### 4.2. Electrophysiological Data

#### 4.2.1. Study Phase

The main focus of this research was the investigation of the study phase in an associative recognition memory task by the means of ERPs in order to identify the eventual different strategies employed by schizophrenia patients compared to healthy controls while encoding the stimulus. The behavioral results obtained in both our present and also previous study [17] indicated that all participants benefited from semantic coherence to increase their recognition performances, as mentioned earlier. However, since behavioral results alone cannot elucidate how semantic coherence was processed in schizophrenia patients versus healthy controls, in the present study, semantic coherence was divided into its external and internal components and analyzed separately by the means of ERPs, using the N400 paradigm. As it was hypothesized, schizophrenia patients showed the N400 external coherence effect, in a similar way as healthy controls did. In other words, patients processed the semantic relationship between the category name and the stimulus, in accordance with behavioral results obtained during the study phase which indicated that patients successfully identified the word pairs as having none, one, or two related words (related to the category name). Indeed, patients strictly observed the instructions given for the task and processed the semantic relationship between category names and word pairs. The category name created an explicit and structured semantic context which helped to increase the subsequent recognition performance. There were only 24 categories and therefore same category names were repeating many times, making the context more salient and helping the patients to process the contextual information. This is in accordance with the literature which suggests that, under some conditions, schizophrenia patients are able to consider the context [39], especially when it is structured and explicitly processed [40–43]. However, there is also a well-established literature showing that schizophrenia patients have contextual processing difficulties and semantic processing disturbances indexed by N400 anomalies observed in priming paradigms [44, 45]. Furthermore, in the study carried out by Green and collaborators [46] who investigated the N400 component of ERPs during the study phase of a recognition memory task with schizophrenia patients, the authors found reduced N400 amplitudes in the patient group compared to healthy controls for words categorized as pleasant or unpleasant at study, indicating N400 abnormalities during episodic encoding. In line with this literature and as hypothesized, contrary to healthy controls who processed both the external and the internal semantic coherence, patients did not process the internal semantic coherence (semantic coherence between the two items within word pairs) as indexed by the lack of N400 internal coherence effect. Since the internal coherence is between the first and the second word, when it is processed, the first word provides the context, and the second word becomes the target. Contrary to the external coherence where the context repeats itself, for the internal coherence, the context word is always unique and each time different. Furthermore, during the study phase, the instructions given to the participants explicitly ask to check the semantic relationship between the category name and the word pairs focusing the treatment on external semantic coherence, without explicitly requiring the treatment of internal coherence. Therefore, patients strictly followed the instructions without initiating any further strategies, whereas healthy controls, in addition to the external coherence, processed also the internal coherence, even if it was not explicitly required by the task instructions. Indeed, the participants knew that, during the test phase, they had to base their old/new answers on the word pairs and not on individual words, and, consequently, it was necessary to process the link between the two items within the word pair during encoding in order to be able to decide, during the test, if the word pair was old or new. Processing the internal semantic coherence during encoding is actually one of the best ways to treat the link between the two items within the word pairs. Healthy controls were able to initiate this strategy during encoding even though it was not explicitly demanded by the task instructions, whereas schizophrenia patients seem to have exclusively followed what was explicitly asked without initiating a strategy which would subsequently be helpful. This pattern of the results with the lack of N400 internal coherence effect accompanied by the presence of N400 external coherence effect is corroborated by studies indicating that schizophrenia patients have strategic memory processing deficits [47] with difficulties to generate effective mnemonic strategies during encoding [48, 49]. However, schizophrenia patients are able to use effective strategies when they are explicitly provided by the task instructions [50]. This indicates that strategy training by the use of clear and explicit instructions could be helpful in the development of cognitive remediation techniques for schizophrenia patients.

#### 4.2.2. Test Phase

Electrophysiological results obtained during the test phase of the present study indicated that semantic coherence induced an FN400 old/new effect in both healthy controls and schizophrenia patients and a late parietal old/new effect in healthy controls alone. The processing of external semantic coherence was accompanied by the subsequent FN400 old/new effect in both groups, while the processing of internal coherence in healthy controls was followed by the late parietal old/new effect in this group of participants alone. Furthermore, the N400 external coherence effect seemed to be related to the subsequent FN400 effect observed in schizophrenia patients during test, whereas, in healthy controls, the N400 external coherence effect was related to the parietal old/new effect.

During the test phase, new word pairs induced more negative amplitudes compared to old word pairs in both groups of participants, which is in accordance with the literature showing that semantic coherence can enhance the early mid-frontal old/new effect in healthy controls [12] and extends this finding to schizophrenia patients, too. The FN400 has been traditionally linked to familiarity. It was however recently suggested that this component could be linked to implicit conceptual memory processing [51]. On the other hand, FN400 and N400 might also be linked to a similar process [23]. Finally, it has been proposed that FN400 might index a more general process concerning implicit contextual facilitation [52]. Therefore, whatever it refers to, the presence of an FN400 old/new effect during test coupled by the presence of N400 external coherence effect during encoding in both groups of participants supports the suggestion of a link between the effective processing of external semantic coherence during encoding and the FN400 old/new effect during test. Furthermore, the correlation analyses showed a positive correlation between the N400 external coherence effect amplitudes and the FN400 old/new effect amplitudes in schizophrenia patients, reinforcing the idea of a relationship between the processing of external semantic coherence and the FN400 old/new effect, at least, in schizophrenia patients. Moreover, the normal modulation of FN400 for semantically related word pairs in schizophrenia patients in comparison to healthy controls might also indicate that patients benefit from the contextual information provided at encoding.

Apart from the FN400 component, test phase results showed a late parietal old/new effect for the related stimuli condition in healthy controls alone. The parietal late positivity (LPC) is considered as an index of recollection process with more positive amplitudes for old stimuli compared to new stimuli during recognition memory tests [26]. However, in our study this pattern was exactly opposite to the expected one with more positive amplitudes for new stimuli than the old ones. This might be in relation to the nature of our new stimuli. Indeed, our new word pairs always contained an old and a new word. As it was suggested by the literature, P600 can be an index of the additional effort necessary for the accomplishment of the task or the revision of the mental representation [53]. The amplitude variation in late positive component may also refer to the cost of reprocessing [54]. In this view, the presence of an old word within the new word pair creates an ambiguity and increases the difficulty to process the new item. This difficulty can be indexed by an increase in the amplitude of late parietal positivity in relation to the cost of reprocessing. Therefore, this difficulty or ambiguity seems to be detected only by healthy controls for semantically related word pairs, while schizophrenia patients would be insensitive to the detection of the so-called anomaly within new word pairs. On the other hand, an increase in the amplitude of late parietal positivity for new items was also observed in the study carried out by Olichney and collaborators [55]. Their study showed, during a categorization task, a word repetition effect for congruous words with a late positivity that was larger for new than old words. This was interpreted by the authors as an index of successful memory encoding. In a similar way, the late parietal old/new effect observed in our healthy controls might be in relation to the successful processing of both external and internal coherence during encoding in this group of participants. If the late parietal old/new effect observed in the present study is a reliable index of recollection, the successful processing of external and internal semantic coherence during encoding in healthy controls could be interpreted as a facilitator or a prerequisite for a successful recollection process during test. Consequently, the presence of both N400 external and internal coherence effects during encoding accompanied by a late parietal old/new effect during test in healthy controls alone might indicate the presence of a link between the processing of item-context and interitem relationships during encoding and a successful recollection at test. It was suggested by the literature that recollection is associated with item-context binding [56] and observed following correct interitem associations [19], since recollection has greater sensitivity than familiarity to interitem associations [57, 58]. Besides, correlation analyses showed a link between the processing of external coherence effect during encoding and the parietal old/new effect during test in healthy controls, which also indicates the presence of a relationship between the item-context binding at encoding and recollection process at test, in healthy controls. However, the internal coherence effect was not correlated to electrophysiological patterns elicited during test. Therefore, interitem binding and recollection might be associated in other ways undetected by the correlation analyses of this study.

Literature showed that early mid-frontal and late parietal old/new effects could be dissociated under different encoding conditions. The parietal old/new effect was found subsequent to deep semantic encoding, whereas the frontal old/new effect was insensitive to the depth of study [59]. Accordingly, deep semantic encoding could lead to both familiarity and recollection based recognition [60], while shallow encoding would lead to familiarity based recognition alone in healthy participants. Hence, deep encoding employed in our study, when combined with the processing of external and internal coherence, may be the reason behind the late parietal old/new effect observed for semantically related word pairs in healthy controls.

The lack of late parietal old/new effect in schizophrenia patients is indeed in line with most behavioral findings of literature showing that recollection process is impaired in schizophrenia [9]. However, relatively few studies investigated recognition memory processes in schizophrenia by the means of ERPs, while studies which focused on ERP old/new effects indicated mitigated results, showing sometimes preserved ERP old/new effects overlapping the LPC in schizophrenia patients [61]. Consequently, the findings of our study provide electrophysiological evidence of recollection impairment in schizophrenia accompanied by semantic processing difficulties at encoding.

#### 4.2.3. Limits of the Study and Conclusion

In our study, the lack of traditional memory measures such as the Wechsler Memory Scale [62] was a major limitation, since memory deficit can be so profound in schizophrenia patients that experimental deficits found can be confounded with this more general memory impairment [63]. However, the fact that our schizophrenia patients were able to normalize their recognition performances for related stimuli makes the presence of a massive and generalized memory deficit less plausible as the reason behind the experimental memory impairment observed in this study. Furthermore, even though the education level of schizophrenia patients was lower than that of healthy controls, they had relatively high education level, and their verbal IQ level was similar to that observed in healthy controls. Literature shows positive correlations between educational level and performances on memory tasks [64–66] on one hand, and the IQ level and memory performance on the other hand [67]. Therefore, the lack of traditional episodic memory measures should not hamper the reliability of our results.

Another point was that our healthy controls had higher education level than schizophrenia patients as it has been just mentioned, and this difference might be a problematic issue. However, since the typical onset of schizophrenic illness during adolescence or early adulthood interrupts the educational attainment, patients tend to have lower education levels than well-matched healthy controls. Therefore matching the groups on the basis of education level may create a “matching fallacy” [68] in which underperforming healthy controls and atypically highly educated patients are recruited. For this reason, in this study, we opted for matching the groups on the basis of their verbal IQ level rather than their education level. Nevertheless, in order to check the impact of education level on different dependent variables of the study, we ran correlation analyses between years of education and behavioral and electrophysiological variables as it was explained in the statistical analyses section. The only significant result was a positive correlation between years of education and correct response rates for new related word pairs during test, while education level did not seem to have any relationships to the rest of experimental scores. Moreover, ANOVA conducted on behavioral results indicated that new words pairs induced similar scores in both groups. There was no effect of coherence or of group by coherence interaction for new word pairs. Thus, even if years of education had a significant correlation with correct response rates for new related word pairs it did not lead to any behavioral impairment. Consequently, we can safely think that the difference observed between the education levels of the two groups does not hinder the solidity of our findings.

In conclusion, our results indicate that schizophrenia patients were able to process the external semantic coherence which was explicitly asked by the task instructions, without however being able to implement any further strategies to process the internal semantic coherence. In other words, patients strictly followed the given instructions without spontaneously generating any strategies which would subsequently be helpful during test. Therefore, the lack of N400 internal coherence effect in schizophrenia patients may indicate the interitem binding deficit during encoding. This is in accordance with the literature suggesting that episodic memory dysfunction in schizophrenia may result from a deficit in relational processing of information during encoding, followed by a recollection deficit during retrieval [69, 70]. Thereby, recollection deficit of patients in associative recognition memory tasks can be related to the lack of interitem relationship processing during encoding, even though correlation analyses in this study did not detect a relationship between the N400 internal coherence effect at encoding and the parietal old/new effect at test. Explicit requirement of this strategy should be tested for the enhancement of recollection in schizophrenia. Within a broader perspective, explicit instructions for the implementation of strategies necessary to the successful accomplishment of the cognitive task in hand could be promising in cognitive rehabilitation of schizophrenia patients.

Furthermore, our results indicate that familiarity process is mobilizable in schizophrenia patients when appropriate semantic strategies are taught during encoding. As it is shown by the literature, strategy training is used in the context of sociocognitive remediation [71] with the objective of bringing patients to self-initiate those strategies in different daily life situations. Hence, cognitive rehabilitation therapies targeting the implementation of semantic encoding strategies by the use of explicit instructions to process the link between the item and the context at encoding, as well as by the reinforcement of the context during both encoding and retrieval, can mobilize the familiarity process which in turn can overcome the recollection deficit in schizophrenic patients. Enhanced familiarity can promote successful episodic memory performance which might be contextualized and integrated into everyday activities in individuals suffering from schizophrenia, allowing them a better functional outcome.

## Figures and Tables

**Table 1:** Sociodemographic and clinical data in the two groups of participants.

	Healthy controls (*n* = 15)	Schizophrenia patients (*n* = 18)	*P* value
Age	28.8 (11)	34.5 (11)	0.156
Years of education	15.6 (1)	14.4 (1)	0.022
Gender (% male)	66.6	55.5	0.515
Verbal IQ	105.2 (8)	105.3 (10)	0.984

PANSS			
Positive/		14 (2)	
Negative/		14 (3)	
General psychopathology/		23 (4)	

Duration of illness (years)		15 (12)	
Chlorpromazine equivalence (mg/day)		497 (637)	

*Note*. Mean scores and standard deviations within brackets.

**Table 2:** Mean correct response rates and standard deviations for each word pair and coherence condition in healthy controls and schizophrenia patients.

Condition	Healthy control	Schizophrenia
Old pair		
Semantically related	0.89 (0.07)	0.84 (0.11)
Semantically unrelated	0.74 (0.17)	0.53 (0.27)

New pair		
Semantically related	0.82 (0.14)	0.65 (0.30)
Semantically unrelated	0.79 (0.09)	0.79 (0.16)
